# Circulating Tumor Cells in Oral Cancer

**DOI:** 10.7759/cureus.51684

**Published:** 2024-01-05

**Authors:** Yashika K Sharma, Madhuri Gawande, Amit Reche, Muskan R Bardia

**Affiliations:** 1 Oral Pathology and Microbiology, Sharad Pawar Dental College, Datta Meghe Institute of Higher Education and Research, Wardha, IND; 2 Public Health Dentistry, Sharad Pawar Dental College, Datta Meghe Institute of Higher Education and Research, Wardha, IND

**Keywords:** treatment implications, metastasis, liquid biopsy, diagnostic tool, biomarkers

## Abstract

Till now, oral squamous cell carcinoma (OSCC) is graded as well-differentiated, moderately-differentiated, poorly-differentiated, and undifferentiated. However, this grading does not have a prediction of the prognosis of the patient. Also, prognosis impacts lymph node metastases, surgical margins, and vascular invasions (neural invasion, muscular invasion, salivary gland invasion). The prognosis of lymph node metastases is significant, which affects the survival of the patients which is 50%. So, a dependable blood marker is needed for prognosis in OSCC patients with loco-regional and distant recurrence. Some factors can be assisted only after surgery and invasive techniques to check the prognosis of the disease. Despite the ease of examining the oral cavity, there is no practical approach for non-invasive screening and detecting cancer. As it is abrupt to use such invasive procedures from time to time, there is a need for nonsurgical and reliable techniques to assess the progression of tumors. Also, frozen sections are helpful during the intraoperative procedure to evaluate the lymph node metastases. An increase in the number of tumor cells through blood is a significant event in disease metastases toward the peripheral blood. Oral health impact assessment instruments could aid in determining the quality of life, and their usage in the initial stages of oral carcinoma could help physicians choose the best treatment option for enhancing the quality of life.

## Introduction and background

More than 90% of all oral malignancies are squamous cell carcinoma. One of India's most prevalent oral cavity cancers is squamous cell carcinoma. Risk factors for oral squamous cell carcinoma (OSCC) include tobacco use (smoking and non-smoking), betel nut chewing, drinking excessive amounts of alcohol, having a human papillomavirus infection, having a poor diet, and not practicing proper dental hygiene. These variables frequently interact, highlighting the significance of prevention through dietary adjustments and routine dental examinations [1].

The widespread use of tobacco and betel quid, strongly ingrained cultural customs in several sections of the nation, is mostly to blame for the high frequency of OSCC in India. Additionally, drinking, especially when combined with tobacco use, is an addiction to the disease burden. Squamous cell carcinoma of the mouth is a malignant squamous epithelial tumor that arises from oral mucosa and has squamous differentiation. In India, OSCC ranks first among males and fourth among females. Men are more prone to OSCC than women, with a ratio of (1.7:1) because men have a higher prevalence of tobacco and alcohol use. The survival rate is 50% despite significant advancements in OSCC diagnosis and therapy. This is due to metastasis and recurrence of malignant OSCC [1-3].

Cancer cells that have broken away from the original tumor and infiltrated the circulation or lymphatic system are known as circulating tumor cells (CTCs) [4]. Tumor cells can split off from the main tumor mass as cancer progresses and travel via lymphatic or circulatory channels to other areas of the body, where they may develop into secondary tumors. Primary tumor releases circulate tumor cells into the bloodstream or lymphatics, providing a non-invasive substitute for conventional cancer diagnosis techniques and reducing the requirement for invasive biopsies. These cells are important markers for the onset of cancer, the effectiveness of treatment, and the potential for metastasis. Researchers and medical professionals can learn a great deal about the metastatic cascade by examining CTCs, which helps them make well-informed decisions on patient therapy. Tracking changes in CTCs over time helps evaluate the efficacy of treatments and the course of the disease, which makes cancer care more individualized and efficient. CTCs contain antigenic and genetic features that are unique to tumors. Because CTCs are more accessible to collect than tissue biopsies and sampling may be repeated, real-time monitoring of patients' treatment responses and metastatic development may be possible [4].

As surrogate markers for overall survival, risk of relapse, and progression-free survival in a variety of carcinomas, particularly in metastatic breast, prostate, and colorectal malignancies, the quantity of CTCs has been discovered over the past 10 years in these carcinomas [1]. Recent research has demonstrated that CTCs can be detected in various tumor types, including pancreatic, lung, renal, bladder, head and neck, melanoma, lung, renal bladder, and bladder. The cell search system (Veridex LLC, Raritan, United States) is the only platform for detecting CTCs in peripheral blood that the Food and Drug Administration has approved. However, there is much knowledge on the collection and analysis of CTCs. Bone marrow sampling is the conventional treatment for nonsolid tumors like lymphoma and leukemia. However, solid tumors have not been adequately investigated. CTCs are believed to have a brief half-life, which is estimated to be two hours in some tumors [3].

This elimination mechanism is aided by fluidic turbulences, shear forces, apoptotic and immune processes, and other difficult circulation conditions. Circulating tumor microemboli (CTM) are collections of tumor cells that contain at least two tumor cells. These clusters have a variety of cell kinds. These microemboli are thought to include groups of migrating cells that intravasate through leaky arteries into the underlying tumor. There is mounting data that suggests the presence of CTMs is a sign of increased metastatic potential. CTCs in the blood have a vital role in the spread of this disease. These cells are non-genetic blood cells and have the genetic properties of various tumors. CTCs can be identified using different approaches, which offer signs of future metastasis [5]. CTCs may reveal new evidence on cancer biology and metastasis in OSCC. As a result, early recognition of metastasis is necessary for a better diagnosis and longevity. The primary tumor releases CTCs, which circulate in the body. Circulating tumor DNA (ctDNA) is a term used to describe the DNA lost by dying tumor cells during necrosis or apoptosis (ctDNA).

Applications of CTCs are pathology screening, diagnosis of disease, malignancy monitoring, and therapy guidance. For the diagnosis, prognosis, and monitoring of cancer treatments, CTCs can be beneficial biomarkers. In OSCC, CTCs can act as prognostic indicators. The existence and measurement of CTCs in the blood may offer important information regarding the disease's severity and propensity for metastasis, aiding in predicting patient outcomes and directing treatment choices. CTCs can be used to track OSCC disease development and treatment efficacy. The success of therapeutic measures can be determined by changes in CTC levels over time, and they can also be used to spot early symptoms of illness return. When neoplastic cells break away from 1º tumor and reach the circulation or lymph system, metastasis occurs. CTCs sow the seeds for metastasis, triggering a cascade that leads to oral cancer-related death. CTCs are shed into the vasculature by a primary tumor and then circulate until they implant at distant places [6]. Recent cancer study findings indicate that CTCs are formed from original tumor clones. Measurement of tumor cells flowing through blood or lymphatic channels may offer definitive proof of metastasis. Moreover, we can diagnose the severity of the condition by detecting CTCs. Regarding technology, it is challenging to detect CTCs since they are common in low concentrations of cells amid a background of countless blood cells. For identification, therefore, susceptible and precise techniques are needed. CTC detection typically involves two steps. An enrichment step is the first step, followed by a detection step. Using their morphological and biological traits, CTCs can be distinguished from other blood cells. Physical enrichment techniques use cell size, density, electric charge, and deformability to isolate CTCs without labeling them (label-free). Techniques for biological enrichment rely on the expression of surface proteins, vitality, and invasion potential. After that, CTCs are found immunocytologically using cytokeratin antibodies [4].

The objective of this article is to conduct a thorough investigation into the function and clinical importance of CTCs in oral cancer. Our goal is to offer a thorough grasp of the processes that lead to CTCs spreading throughout patients with oral cancer, their potential as prognostic and diagnostic indicators, and their importance in directing therapeutic approaches. This article aims to enhance personalized treatment for patients with oral cancer by presenting new insights and combining current research findings. This will ultimately improve oral cancer patients' early detection, care, and overall outcomes.

## Review

Methodology

**Figure 1:**
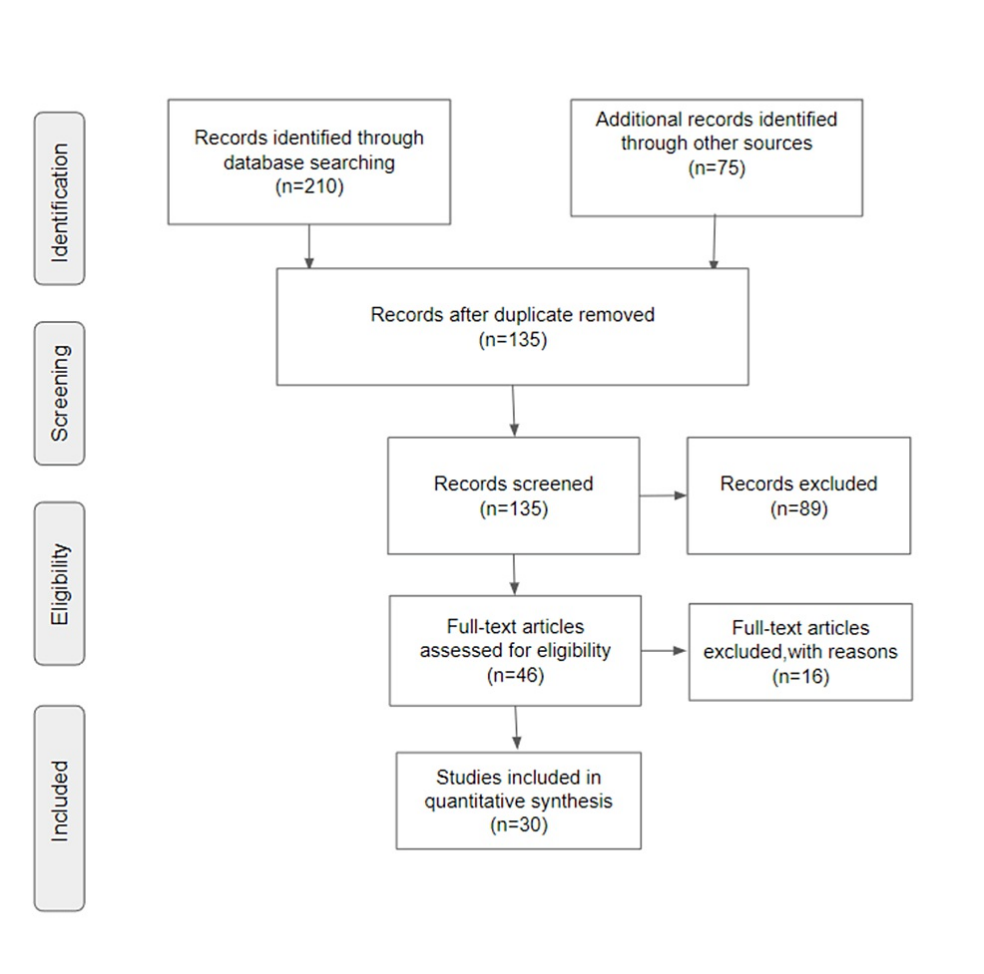
PRISMA flow diagram for search strategy Adopted from PRISMA. PRISMA: Preferred Reporting Items for Systematic Reviews and Meta-Analyses

This review carefully gathered literature on CTCs in oral cancer utilizing electronic databases such as PubMed, Scopus, and Web of Science. Keywords like "biomarkers," "diagnostic tool," "liquid biopsy," "metastasis," and "treatment implications" were used to search the database. Articles on CTCs in oral cancer were included, but publications focusing on CTCs in oral cancer in other disciplines were omitted. The inclusion criteria included relevant books, articles, studies, conference presentations, gray or unpublished literature, and reviews. The study selection procedure included screening titles and abstracts, followed by a full-text evaluation of relevant papers. The final group of included research offers a thorough analysis of the evidence that is currently available on CTCs in oral cancer. The results were combined and analyzed to draw meaningful conclusions. Figure 1 describes the selection process of articles used in our study.

Cancer patients may have CTCs in their bloodstream or bone marrow known as disseminated tumor cells (DTC). It is believed that the bone marrow contributes significantly to the growth of tumor cells by providing a feeding environment. Improved staging methods for OSCC are needed to detect early metastatic dissemination and residual tumor presence. As a result, better therapeutic intervention decisions are essential. However, recently released data reveals that DTCs and CTCs are distinctive prognostic markers of cancer-free survival in patients with OSCC. The frequency of distant metastasis has dramatically increased even though lymph node metastasis in the neck is the most significant prognostic indicator for relapse. Despite significant improvements in diagnosis and therapy options, the five-year survival rate across all tumor stages has remained constant over the previous few decades at around 50% [5]. This discovery could result from the clinical disease's altered pattern. Tumor cells develop "cell plasticity" and split off from the main tumor site after undergoing a partial or total change from an epithelial to a mesenchymal phenotype. A rupture of the basement membrane and intravasation into the bloodstream or lymphatic system occurs next. Certain tumor cells cannot survive in circulation because of rapid velocity, shear bidirectional venous and aortic stress, and immunological surveillance. Apoptosis may occur in some cells. The surviving cells may continue to be dormant or, by forming micrometastasis, may progress into secondary, overt macrometastases in distant regions. CTCs can move as single cells and in cell clusters known as CTM, which have a more significant metastatic potential [4].

CTC detection methods

**Figure 2:**
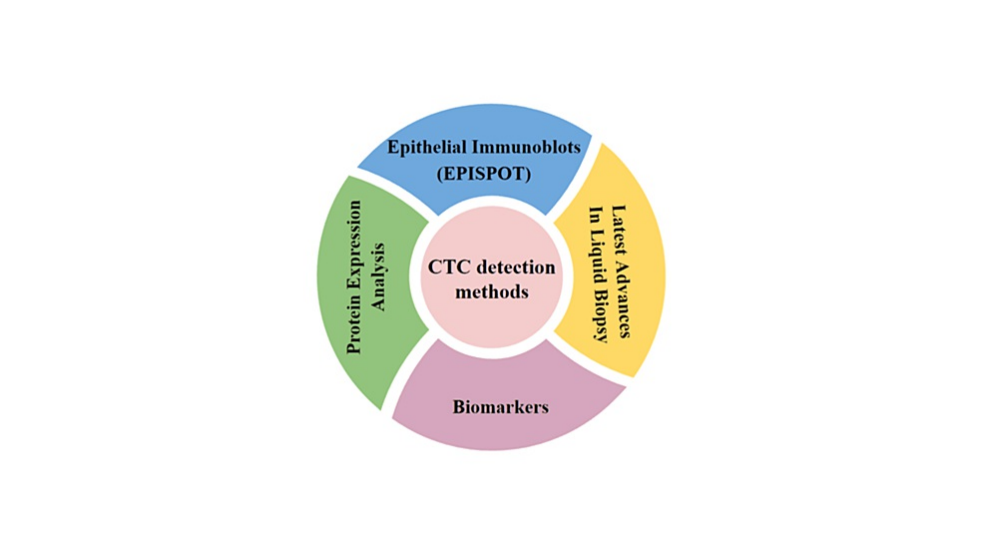
Various methods for detection of circulating tumor cells (CTC)

Several methods for detecting the rare CTCs have been developed. To put the rareness of these cells into position, even in metastatic disease, it is believed there may only be one tumor cell amid crores of other cells using currently available technologies. CTCs are detected and analyzed using a variety of approaches by researchers and physicians, including immunomagnetic cell separation, microfluidics-based devices, and polymerase chain reaction procedures. An initial enrichment step is required to improve the detection effectiveness of these cells. Basic enrichment approaches rely on translation related to specific molecules gathered by magnet-beaded coat antibodies or physical characteristics such as shape, volume, and dielectrophoretic movement, which may be considered CTC sub-groups [7]. After the depletion of blood cells, tumor cells are extracted in an initial enrichment stage. The tumor cells are labeled or tested for oncogenes. Antibodies are used to mark CTCs, and DNA primers are used to probe them. Cytometry, microscopy, conductometry, fiber optics, reverse transcription-polymerase chain reaction, fluorescence in situ hybridization, and relative genomic hybridization are all used to detect tumor cells. CTCs are enriched regarding the physical qualities, such as mass, volume, dielectrophoretic motion, or the expression of specific cell surfaces identified via magnet bead-coated antibodies [8]. The Flow Cytometry Software (FCS) express program quantitatively evaluated Nanog protein-expressing cells using a murine anti-human antibody coupled with fluorescein isothiocyanate, a primary derivative. Figure 2 describes various CTC detection methods.

Protein Expression Analysis

CTCs are found in extremely little concentration in blood, as less as one CTC per crore of RBC. As a result, a key challenge is to recognize these CTCs with great sensitivity and specificity amid billions of other cells. Traditional approaches for detecting CTCs based on histological investigation are time-consuming and inaccurate [9]. The potential of CSCs to produce micro-spheres is used in cellular-based evaluation techniques. Following 48 hours of cultivation, we examined the micro-sphere cheerful populace of Roswell Park Memorial Institute (RPMI)-1640-cultivated molecules and STEMPRO-cultivated cells depending on their tendency to construct spherical groups in semi-suspension using human cancer stem cell cultures as a positive control. For inspection and evaluation, a light microscope was employed.

Epithelial Immunoblots

Epithelial immunoblots (EPISPOT) can detect live CTCs after producing particular proteins during a 48-hour short-term culture. Leukocytes must be eliminated before employing the EPISPOT method, which requires adverse selection of hemopoietic cells, such as when CD45, a common leukocyte antigen, is used. Significance of liquid biopsy in oral cancer - as the concept of solid tumors as a systemic disorder has gained hold, CTC and DTC in the periphery of the blood/bone marrow of diseased patients have been proven to be extremely important [4]. Applying separation technologies has greatly impacted the morphological and genetic investigations of CTCs [10]. Tumor cells lose epithelial markers after metastasis, making CTC detection harder. As a result, evaluating the presence of telomerase would aid in compiling a picture of telomere activity. The biggest drawback of this approach is that it requires a complete blood sample. The sample must be lysed to determine the enzymatic activity, and as a result, the CTCs are destroyed as well [11]. CTCs can be separated without a labeling number of techniques, such as density gradient centrifuge and purification with specialized filters. This label-free bio-chip can determine differences in cancer cell shape and malformation, a photoacoustic Fluorescence-activated cell sorting (FACS), and the di-electrophoretic separation method. Although the current CTC technologies have not yet been fully adopted, they show great potential as future clinical diagnostic methods for the tailored treatment of cancer patients [12-14].

Increasing the number of CTCs: These procedures supply CTCs from ex-vivo samples. However, a recent innovation permits in vivo nuritioment of CTCs directly into a patient's forearm vein utilizing a "GILUPI nano detector." CTC is enriched; roughly one and a half liters of blood (containing CTC) flow through the nano detectors at about half an hour of use in a centimeter functional area [15]. When lump-specific antigens were investigated, recent research revealed that circulating tumor DNA (ctDNA) was a better predictive marker than CTC. In recent studies, circulating cell-free DNA has been proven to be so sensitive in picturizing the entire malignant condition that it can be reliably used as a source of DNA to help us detect the malignancy, substituting tumor tissue in established diagnostic procedures [16]. Following studies on the dissemination of cancer cells post-surgery and the increase of tumor cells as a result of surgical treatments for OSCC patients, only a few of these studies have looked into the influence of metastasis and survival in this regard. Because the present gold standard and primary treatment option is surgery for OSCC, these facts are critical [17-18].

Latest Advances In Liquid Biopsy

Exosomes of nearly 3020 nm can be extracted from various biofluids, including saliva plasma serum cerebrospinal fluid urine. Tumor exosomes can promote tumor cell proliferation, inhibit immunity, and initiate the formation of new blood vessels and accumulation in distant tissue. Exosomes are primarily released through two mechanisms: one involves the formation of multivesicular bodies, and the other entails the spontaneous budding of exosomes from cellular membranes. Exosomes, secreted by every cell, contribute to the detection of mutations as well as the RNA profiling of many diseases, such as cardiovascular and neurological diseases. Exosomes, for example, CTC, carry membrane markers from their source cell and can be enhanced with the same technique. Exosomes can be isolated and sorted from bodily fluids using nanoscale fluorescence-activated cell sorting [19].

Biomarkers

To find particular molecular biomarkers linked to OSCC, CTCs might be examined. Understanding the genetic and molecular properties of CTCs may help identify possible treatment options and the biology behind the disease. It is crucial to find suitable patients for individualized therapy and to regularly measure CTC levels in each patient to monitor their progress. Human epidermal growth factor receptor 2 expression on CTCs can be tracked and is a potential marker for breast cancer. Unfortunately, there isn't a biomarker for OSCC yet. By considering DTCs as therapeutic targets, BM might be a fascinating target organ for therapeutic interventions. The information provided here suggests that medications that target DTCs may one day help treat OSCC patients' BM. When it is established that these techniques are appropriate for OSCC and can be incorporated into established protocols for tumor staging, the enormous costs that are currently spent will decline. Identifying CTCs is critical to clinical oncology and cancer research. A non-invasive method for tracking the course of a disease, evaluating the effectiveness of a treatment, and maybe forecasting patient outcomes is the identification and quantification of CTCs. This study provides an in-depth investigation of the most recent detection techniques used in CTC analysis [9]. These methods cover various tactics, including molecular, physical, and immunological methods. The paper explores the complexities of modern molecular assays, microfluidic devices, and immunomagnetic separation, offering a thorough evaluation of each technology's benefits and drawbacks. We also examine new technologies that have the potential to improve sensitivity and specificity, like single-cell analysis and label-free techniques. This article also addresses the difficulties in detecting CTCs, including their blood sample scarcity and demographic variability. It looks at the methods used today for CTC separation and enrichment and stresses the value of following defined procedures to guarantee reliable and repeatable outcomes. It also describes how machine learning and artificial intelligence can be integrated for CTC detection and classification, highlighting how this could transform CTC analysis. In conclusion, this study highlights the vital need for ongoing developments in this area and thoroughly reviews the changing landscape of CTC detection techniques [10].

Discussion

In a research carried out by Saucedo-Zeni about OSCC victims, it was evident that early diagnosis of CTCs will, at a specific rate, increase the existence and kind of life in OSCC victims, emphasizing the importance of early detection [20]. In a study by Sharma and colleagues using two separate primer sets, the recorded data revealed the expression pattern of Ck19. Pre-biopsy, pre-surgery, and post-surgery after three months, the appearance of Ck 19 was analyzed, and specific samples showed a massive rise in the rate of Ck19 three months post-operation when differentiated to pre-biopsy/surgery in both cases. The presence of Ck19 in the CTCs increases the probability of detecting metastasis and regional recurrence in OSCC patients [21]. Saliva has a hopeful association with OSCC planning and monitoring; new studies about the use of circulating tumor DNAs, extracellular vesicles, microRNAs, and CTC as saliva biomarkers in OSC routine practice may aid in the growth of consistent strategies for initial tumor detection in lesions, promote prevention, and assist the development of therapy; it will enhance clinical outcome. The clinical usefulness of saliva for diagnosing systemic disorders, including malignancies, has been validated in several recent papers [22]. Alix-Panabie used body fluid samples from SCC of HN patients, which were nutrition for CTC by FACS measurement of CD-45, to investigate the relationship between CTC and lymphatic node progression in inoperable malignancies of the head-neck area. As a result, this study concluded that CTCs are associated with local metastasis in the non-resectable SCC of HN that research would assist in determining the "prognostic importance" of the CTCs identification in conjunction with the clinical lymph node grading for local or distant recurrence [23]. In a news briefing at the "American-Association-for-the-Advancement-of-Science" Annual Meeting 2016, Parkinson discussed a device called electric field-induced release and measurement (EFIRM). This device requires a few drops of saliva. The test might take as little as ten minutes to complete. The device is thought to identify epidermal-growth-factor-receptor-genetic-alterations (EGFR) [24]. Wikner and his colleagues discovered several "signature mutations," such as PIKI3CA, FGFR3, EGFR, Double Data Rate 2, PTEN, TP53, and others, which account for more than 75% of HPV-related oropharyngeal malignancies. As claimed by the professor, detecting these mutations through saliva screening may provide physicians and patients with room to maneuver for early diagnosis and therapy for cancers with a five-year survival probability of around 60% [25]. The circulating tumor stem-like cells CD44 and Nanog have been identified as markers for assessing local and regional aggressiveness, detecting relapse, and responding to therapy. This study by Powell examined the possible role of these markers in OSCC. On the other hand, there are now more uncertainties than answers about the ability to characterize the genetics of CTCs. The levels of 95 cancer-related genes were examined in CTCs from 50 breast cancer patients last year, and the results showed that different patient cells, even from the same patient, had different gene expression patterns [26]. According to Chen, more study is required to understand the connection between a patient's tumor and CTCs and ascertain the clinical utility of CTCs [27]. There are already many different CTC detection technologies available. However, according to Lin, their sensitivity and specificity still need to be increased. A new age for CTC analysis and clinical applications has emerged with epithelial marker-based CTC detection technology, such as the cell search system. However, researchers are quickly realizing and appreciating its shortcomings [28]. However, it is interesting to note that recent research by Mong has shown that the cells from cancer patients' non-cancerous tumors are also significant surrogate biomarkers for cancer patients [29]. The majority of CTC research to date has concentrated on CTCs in the bloodstream. Ashworth initially reported CTCs in 1869 after observing "some cells" in a metastatic cancer patient's blood that resembled tumor cells from the original tumors [30]. A summary of all the articles included in this review is listed in Table 1.

**Table 1:** Summary of the articles included in the review CTC: Circulating tumor cells; SCCHN: Squamous carcinoma of head and neck region; DNA: Deoxyribonucleic acid; OSCC: Oral squamous cell carcinoma

Author	Year	Findings
Shah JP and Gil Z [1]	2009	Explores contemporary approaches to surgical management of oral cancer.
Attar E et al. [2]	2010	Presents a population-based perspective on head and neck cancer in a developing country.
Bagan J et al. [3]	2010	Focuses on the clinical features of oral cancer.
Mollaoglu N et al. [4]	2009	Detecting single disseminated tumor cells in peripheral blood samples of patients with oral squamous cell carcinoma.
Hristozova T et al. [5]	2011	Correlation between circulating tumor cells (CTCs) and lymph node metastasis in non-resectable squamous cell carcinoma of the head and neck region (SCCHN).
Anderson KS et al. [6]	2011	Identifies serum antibodies to the HPV16 proteome as potential head and neck cancer biomarkers.
Brock G et al. [7]	2015	Application of liquid biopsy for cancer screening, patient stratification, and monitoring.
Jatana KR et al. [8]	2011	Circulating tumor cells as a prognostic marker in head and neck squamous cell carcinoma.
Kusukawa J et al. [9]	2000	Dissemination of cancer cells into circulation can occur through incisional biopsy of oral squamous cell carcinoma.
Li YM et al. [10]	2013	Epithelial-mesenchymal transition markers expressed in circulating tumor cells.
Cristaldi M et al. [11]	2019	Salivary biomarkers' current status and perspectives for oral squamous cell carcinoma diagnosis and follow-up.
Heitzer E et al. [12]	2015	Circulating tumor DNA as a liquid biopsy offers insights into its potential applications.
Bankó P et al. [13]	2019	Technologies for circulating tumor cell separation from whole blood.
Patel S et al. [14]	2016	Presence of circulating tumor stem-like cells in oral squamous cell carcinoma.
Borgen E et al. [15]	1999	Standardizing the immunocytochemical detection of cancer cells in bone marrow and blood.
Chowdhury R et al. [16]	2018	Screening and monitoring of oral cancers by detecting circulating tumor cells.
Anitha N et al. [17]	2015	Presence of circulating tumor cells in oral squamous cell carcinoma.
Bagri-Manjrekar K et al. [18]	2018	Correlates the in vivo autofluorescence of oral squamous cell carcinoma to the cell proliferation rate.
Alix-Panabières C et al. [19]	2013	Circulating tumor cells as a liquid biopsy for cancer, providing insights into their diagnostic potential.
Saucedo-Zeni N et al. [20]	2012	A novel method for the in vivo isolation of circulating tumor cells from the peripheral blood of cancer patients using a functionalized and structured medical wire.
Sharma P et al. [21]	2012	Microscopy-assisted cytomorphometric analysis of oral exfoliated cells in OSCC.
Kaczor-Urbanowicz KE et al. [22]	2019	Saliva's clinical validity and novel cancer detection technology.
Alix-Panabières C et al. [23]	2012	Circulating tumor cells as promising biomarkers in cancer research and diagnostics.
Parkinson DR et al. [24]	2012	Considerations in developing circulating tumor cell technology for clinical use.
Wikner J et al. [25]	2014	Association between squamous cell carcinoma of the oral cavity and circulating tumor cells.
Powell AA et al. [26]	2012	Single-cell profiling of circulating tumor cells.
Chen H et al. [27]	2017	Highly sensitive capture of circulating tumor cells using micro-ellipse filters.
Lin D et al. [28]	2021	Biology and clinical significance of circulating tumor cells.
Mong J et al. [29]	2018	Size-based enrichment technologies for non-cancerous tumor-derived cells in the blood.
Ashworth TR et al. [30]	1869	A case of cancer in which cells similar to those in the tumors were observed in the blood after death.

## Conclusions

In conclusion, prognosis, treatment planning, and metastasis assessment of OSCC depend greatly on knowledge of the function of CTCs. Progression in research and technology can help pinpoint particular CTC subpopulations, which can yield important information for specialized treatment strategies. The importance of CTCs has been acknowledged, highlighting their potential as a vital instrument for improving OSCC patient outcomes through increased diagnostic accuracy as well as personalized treatment plans. The detection of CTCs and, consequently, the prediction of the patient's prognosis and treatment plan will be facilitated by the standardization of such techniques.
